# Root resorption, treatment time and extraction rate during orthodontic treatment with self-ligating and conventional brackets

**DOI:** 10.1186/1746-160X-10-2

**Published:** 2014-01-23

**Authors:** Collin Jacobs, Philipp F Gebhardt, Viviana Jacobs, Marlene Hechtner, Dan Meila, Heinrich Wehrbein

**Affiliations:** 1 Department of Orthodontics, University of Mainz, Mainz, Germany; 2 Department of Medical Biostatistics, Epidemiology and Informatics, University of Mainz, Mainz, Germany; 3 Department of Diagnostic and Interventional Neuroradiology, Hannover Medical School, Hannover, Germany

**Keywords:** Apical root resorption, Self-ligating, Multibracket appliance, Treatment time

## Abstract

**Introduction:**

This study determined the amount and severity of EARR (external apical root resorption) after orthodontic treatment with self-ligating (SL) and conventional (Non-SL) brackets. Differences regarding rate of extraction cases, appointments and treatment time were evaluated.

**Material and methods:**

213 patients with a mean age of 12.4 ± 2.2 years were evaluated retrospectively. The treatments were performed with SL brackets (n = 139, Smartclip, 3 M Unitek, USA) or Non-SL brackets (n = 74, Victory Series, 3 M Unitek, USA). Measurements of the crown and root length of the incisors were taken using panoramic radiographs. Three-factor analysis of variance (ANOVA) was performed for an appliance effect.

**Results:**

There was no difference between patients treated with Non-SL or SL brackets regarding the amount (in percentage) of EARR (Non-SL: 4.5 ± 6.6 vs. SL: 3.0 ± 5.6). Occurrence of severe EARR (sEARR) did also not differ between the two groups (Non-SL 0.5 vs. SL: 0.3). The percentage of patients with need of tooth extraction for treatment (Non SL: 8.1 vs. SL: 6.9) and the number of appointments (Non-SL: 12.4 ± 3.4 vs. SL: 13.9 ± 3.3) did not show any differences. The treatment time was shorter with Non-SL brackets (Non-SL: 18.1 ± 5.3 vs. SL: 20.7 ± 4.9 months).

**Conclusions:**

This is the largest study showing that there is no difference in the amount of EARR, number of appointments and extraction rate between conventional and self-ligating brackets. For the first time we could demonstrate that occurrence of sEARR does not differ between the two types of brackets.

## Introduction

External apical root resorption (EARR) is defined as either a physiologic or pathologic process with the loss of cementum or dentine resulting in a shortening of the root apex. This process is often associated with orthodontic treatment [1].

Since EARR is a serious iatrogenic problem, there has been intensive research about EARR as an adverse effect during orthodontic treatment. As a result many studies have underlined that EARR often develops during treatment with the fixed multibracket appliance [1-5]. According to this and the fact that mechanical forces are a key factor in the occurrence of EARR, studies have shown, that the appliance or technique used for an orthodontic treatment can be related to the degree of EARR [4,6-8].

Furthermore several other factors have been implicated in the initiation and progression of EARR’s during orthodontic treatment, such as the duration of treatment [9-11], the level of force applied [12,13], idiopathic EARR before treatment [14,15] and the type of movement, e.g. torque, intrusion or bodily movement [8,13,16-23]. In addition a genetic predisposition is assumed. Since orthodontic treatment with a fixed appliance can act as a trigger for severe EARR (sEARR) in genetically predisposed individuals [14], it is estimated, that the proportion of the hereditary component is 60%-80% for EARR [24].

The teeth most affected by EARR are the maxillary and mandibular incisors indicating that mechanical factors might play an important role in the development of EARR [18,20,25,26]. SEARR are present when more than 1/3 of the root is resorpted. With conventional brackets (Non-SL) sEARR occurred in about 0.5% of patients under orthodontic treatment [27].

The search for improved efficiency in orthodontic treatment with less adverse effects has generated new types of brackets [28]. Self-ligating (SL) brackets are an innovation, which have been pioneered in the 1930s. These brackets provide a mechanism of closure for the inserted archwire, so no additional rubber elastics or steel ligatures are needed. Still they have undergone a revival in the recent years with a variety of new features being developed and advanced. A host of advantages such as shorter treatment time, higher rate of tooth movement and fewer appointments have been claimed relating to reduced frictional resistance of these brackets [29].

By now there are a few studies, which observed no significant benefits of SL brackets compared to Non-SL brackets regarding the occurrence of EARR, treatment time or number of appointments. However most of these studies are with a small amount of patients or compare brackets of different companies or with different prescriptions between them [28,30-34]. The occurrence of sEARR regarding SL- and Non-SL brackets has not yet been compared.

The objective of this study was to determine the occurrence and severity of EARR on maxillary and mandibular incisors during treatment with SL and Non-SL brackets with the same prescription and from the same company. We furthermore aimed to analyze the amount of extraction cases, number of appointments and treatment time.

## Materials and methods

### Patients and inclusion/exclusion criteria

For this retrospective study 213 patients, being treated in a private praxis between 2008 and 2012, were included by the following criteria:

Inclusion criteria:

– completed treatment with a multibracket appliance

– presence of panoramic radiograph before and after treatment

– completed root growth of the maxillary and mandibular incisors before treatment

– no evidence of EARR of the maxillary and mandibular incisors on the pretreatment panoramic radiograph

– no severely dilacerated incisor roots

– caries-free maxillary and mandibular incisors

Exclusion criteria:

– impacted teeth

– trauma before and during active treatment

– bends of first, second or third order in the archwire

– multiple agenesis

– need for orthognathic surgery

– endodontic treatment

– fixed class II appliance

### Treatment procedure

139 patients (females n = 83; males n = 56) were treated with SL brackets (Smart Clip, 3 M Unitek, Monrovia, CA, USA). 74 patients (females n = 51; males n = 23) functioned as a control group treated with Non-SL brackets (Victory, 3 M Unitek, Monrovia, CA, USA). Both bracket types provided 0.022 slots sizes and MBT prescriptions. Treatments of all patients were performed by the same practitioner with a general arch-wire sequence of a 0.015 twistflex (stainless steel), 0.016 nickel-titanium, 0.016 × 0.022 nickel-titanium, 0.017 × 0.025 nickel-titanium and 0.019 × 0.025 stainless steel (Resilient Orthoform III OVOID, 3 M Unitek, Monrovia, CA, USA). Ligation in the control group was performed with steel ligatures for the conventional brackets.

### X-ray measurement and patient informed consent

**Figure 1:**
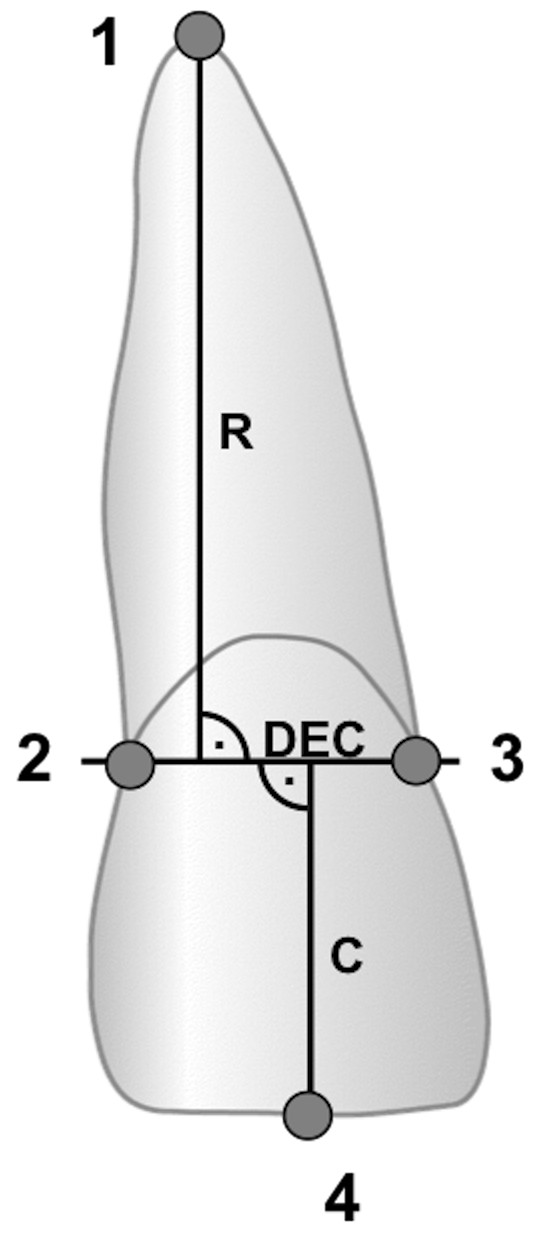
**Intersection for incisors according to Linge and Linge.** Reference points: **1** root apex, **2** distal dento-enamel junction, **3** mesial dento-enamel junction, **4** incisal edge. Dento-enamel conjunction (DEC) represents the conjunction between mesial and distal. Crown length (**C**) and root length (R) were measured perpendicular to DEC as the longest distance to the root apex and the incisal edge.

EARR was defined as any reduction in the radiographic lengths of the maxillary and mandibular incisor teeth from the tip of the incisal edge to the apex of the root. Quantitative measurements of the crown and root length of the maxillary and mandibular central and lateral incisors were taken. Any image distortion between the pre- and post-treatment radiographs was calculated using the crown length registrations. This measurement method was established by Linge and Linge and has been described by several studies [26,27] (Figure 1).

Correction Factor (CF) = C1/C2

C1 = Crown length on pretreatment radiograph

C2 = Crown length on post-treatment radiograph

The EARR in millimeter was calculated as following.

EARR = R1 – (R2 x CF)

R1 = Root length before treatment

R2 = Root length after treatment

It was decided to express the EARR as relative root resorption (rRR) seen as the percentage shortening per tooth.

$$\mathrm{rRR}\;\left(\text{Resorption}\;\text{per}\;\text{tooth}\;\mathrm{in}\;\%\right)=\left(\text{EARR}\times \;100\%\right)/R1$$

**Figure 2:**
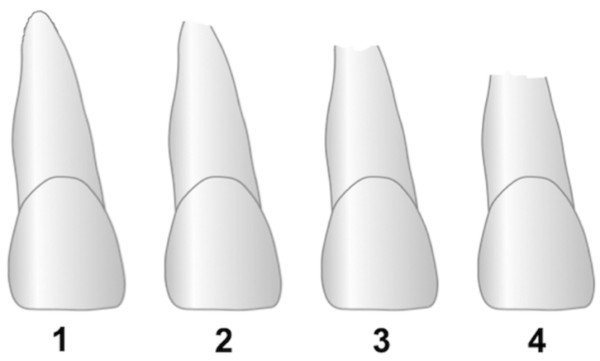
External apical root resorption (EARR) stages according to Malmgren et al. 1 irregular root contour, 2 EARR <2 mm of root length, 3 EARR >2 mm to 1/3 of root length, 4 EARR >1/3 of root length.

A tooth is defined as exhibiting a sEARR, when the grade-IV root resorption according to Malmgren et al. is present [15]. In this case more than 1/3 of the root has been resorbed, for example if rRR > 33.33% (Figure 2).

Pre- and post-treatment radiographs were present due to the usual treatment procedure in Germany, where it is required for diagnostic and treatment procedure. All panoramic radiographs were taken by the same operator. No radiographs were taken out of study reasons and all data were analyzed anonymously to protect the rights of the patients. All patients were informed about using their data for research and gave their informed consent.

### Statistical assessment

Statistical analyses were performed using SPSS 20.0 (SPSS Inc., Chicago IL, USA) and SAS 9.2 (SAS Institute, Cary, NC, USA). All multivariable statistical models were adjusted for gender, age, and treatment duration. The two main research questions comprised:

(1)  The association of SL brackets with EARR. This was investigated using a repeated measures mixed linear model with fixed and random effects. The model was further adjusted for extraction, treatment mechanism, type of tooth, and interaction between tooth location and self-ligating brackets.

(2)  The impact of SL brackets on the occurrence of sEARR. For this we used generalized estimating equations to fit a repeated measures binary logistic regression model.

The global significance level of 0.05 was adjusted using a Bonferroni correction which led to local significance levels of α = 0.025.

Secondary research questions included the association of SL brackets with (a) the number of visits within a treatment, (b) the duration of treatment, and (c) the number of extraction cases. This was analyzed using multivariable ANCOVA models additionally adjusted for extraction and treatment mechanic and a binary logistic regression model.

These analyses can be regarded as explorative and respective p-values as descriptive.

### Error determination

To determine systematic error, each of 10 panoramic radiographs taken before and after treatment were reanalyzed five months after the first assessment, a time period long enough for the original measurements to be forgotten in order to determine statistical error according to Dahlberg’s formula. The error in the panoramic radiographs assessment fell within a very good range of (0.14 mm).

## Results

Two hundred thirteen patients were included into the study, with 139 (65.3%) allocated for treatment with SL brackets and 74 (34.7%) for Non-SL brackets. For the SL group the treatment sample consisted of 56 (40.3%) males and 83 (59.7%) females and for the Non-SL group it consisted of 23 (31.1%) males and 51 (68.9%) females. Overall 1704 teeth were measured before and after treatment (Table 1).

**Table 1:** Patient data

	**SL (n = 139)**		**Non-SL (n = 74)**		**Total (N = 213)**	
	**n (%)**	**Mean ± SD**	**n (%)**	**Mean ± SD**	**n (%)**	**Mean ± SD**
Amount of teeth	1112		592		1704	
Gender						
Male	56 (40.3)		23 (31.1)		79 (37.1)	
Female	83 (59.7)		51 (68.9)		134 (62.9)	
Age at start of treatment (years)		12.6 ± 2.3		12.1 ± 2.2		12.1 ± 2.2

SL: self-ligating, SD: standard deviation.

We observed rRR of 4.5% in the group of patients treated with Non-SL brackets and of 3.0% of patients treated with SL brackets (Table 2). Out of all 1704 examined teeth, 6 teeth (0.4%) developed sEARR. Within the Non-SL group 3 (0.5%) teeth in 3 patients were affected. In the SL group also 3 teeth (0.3%) in 3 patients had developed sEARR (Table 3). The multivariable mixed linear model analysis showed no significant impact of SL brackets on the rRR compared to Non-SL brackets (parameter estimate (β) = -0,86, 95% confidence interval (CI) -2.59-0.87, p = 0,33). SL brackets had also no significant impact on the occurrence of sEARR compared to Non-SL brackets (OR = 0.92, 95% CI 0.24-3.50, p = 0.91) (Table 4).

**Table 2:** Amount of rRR between SL and Non-SL (tooth level)

	**SL (n = 1112)**	**Non-SL (n = 592)**	**Total (n = 1704)**
	**Mean ± SD**	**Mean ± SD**	**Mean ± SD**
rRR (%)	3.0 ± 5.6	4.5 ± 6.6	3.5 ± 6.0

SL: self-ligating, SD: standard deviation, rRR: relative root resorption.

**Table 3:** Percentage of teeth affected by severe EARR (tooth level)

	**SL (n = 1112)**	**Non-SL (n = 592)**	**Total (N = 1704)**
	**n (%)**	**n (%)**	**n (%)**
sEARR			
Present	3 (0.3)	3 (0.5)	6 (0.4)
Not present	1109 (99.7)	589 (99.5)	1698 (99.6)

SL: self-ligating, SD: standard deviation, EARR: external apical root resorption.

**Table 4:** Multivariable analysis evaluating the impact of SL brackets on rRR and sEARR

	**rRR**^**a**^			**sEARR**^**b**^		
	**Parameter estimate (β)**^**c**^	**p-value**	**95% CI**	**OR**^**d**^	**p-value**	**95% CI**
SL	-0.86	0.33	-2.59; 0.87	0,92	0.91	0.24; 3.50
Non-SL	0^e^			0^e^		

^a^repeated measures mixed linear model adjusted for gender, age, treatment duration, amount of extraction, treatment mechanic, location of the tooth, and interaction between tooth location and self-ligating brackets. ^b^repeated measures binary logistic regression adjusted for gender, age, and treatment duration, ^c^regression coefficient: average difference in rRR between the Non-SL (reference group) and the SL group (comparison group), ^d^Odds ratio: chance of sEARR occurring in the SL group compared to the chance of sEARR occurring in the Non-SL group. An OR of 1 indicate equal chances in both groups, OR > 1 indicate higher chance in the SL vs. non-SL group, OR < 1 indicate lower chance in the SL vs. Non-SL group, ^e^Reference category.

CI confidence interval, OR: odds ratio, rRR: relative root resorption, sEARR: severe external apical root resorptions, SL: self-ligating.

Analyzing the locations of the teeth, which were most frequently affected by sEARR, the tooth group 31/41 occurred to have the highest frequency of sEARR (n = 4) (Table 5).

**Table 5:** Number of teeth affected by severe EARR according to tooth group (grade-IV RR according to Malmgren) (tooth level)

	**Teeth affected by sEARR (n = 6)**	**Teeth non-affected by sEARR (n = 1698)**	**Total (N = 1704)**
Tooth group	n (%)	n (%)	n (%)
12,22	1 (0.2)	425 (99.8)	426 (100.0)
11,21	1 (0.2)	425 (99.8)	426 (100.0)
32,42	0 (0.0)	426 (100.0)	426 (100.0)
31,41	4 (0.9)	422 (99.1)	426 (100.0)

EARR: external apical root resorption, sEARR: severe external apical root resorption.

The orthodontic treatment of the group with Non-SL brackets took on average 18.1 ± 5.2 months, the treatment of the group with SL brackets 20.7 ± 4.9 months (Table 6). Thus, the bracket type was seen to influence treatment duration with a statistical relevance. Patients treated with SL brackets received a 2.6 months longer treatment duration than patients with Non-SL brackets (β = 3.52, 95% CI 2.07-4.96, p < 0.001). The Non-SL group had on average 12.4 ± 3.4 visits and the SL group 13.9 ± 3.3 visits. In the multivariable analyses treatment with SL brackets did not impact the number of visits (β = -0.50, 95% CI -1.15-0.16, p = 0.14). Analyzing the amount of extraction cases between the two groups we could not observe a significant difference. In the Non-SL group 6 (8.1%) patients had an extraction compared to 9 (6.5%) patients in the SL group (OR = 0.84, 95% CI 0.26-2.49, p = 0.76) (Table 6).

**Table 6:** Treatment data (patient level)

	**SL**		**Non-SL**		**Total**	
	**(n = 139)**		**(n = 74)**		**(N = 213)**	
	**n (%)**	**Mean ± SD**	**n (%)**	**Mean ± SD**	**n (%)**	**Mean ± SD**
Treatment time (months)		20.7 ± 4.9		18.1 ± 5.3		19.8 ± 5.2
Number of visits		13.9 ± 3.3		12.4 ± 3.4		13.4 ± 3.4
Number of extraction cases (patients)	9 (6.5)		6 (8.1)		15 (7.0)	

SL: self-ligating, SD: standard deviation.

Table 7 shows the data based on a multivariable analysis for the number of visits, the duration of treatment and the extraction cases.

**Table 7:** Multivariable analysis evaluating the impact of self-ligating (SL) brackets on number of visits within a treatment, treatment duration, and number of extraction cases

	**Number of visits**^**a**^			**Duration of treatment**^**a**^			**Extraction**^**b**^		
	**Parameter estimate (β)**^**c**^	**p-value**	**95% CI**	**Parameter estimate (β)**^**c**^	**p-value**	**95% CI**	**OR**^**d**^	**p-value**	**95% CI**
SL	-0.50	0.14	-1.15; 0.16	3.52	<0.001	2.07; 4.96	0.84	0.76	0.29; 2.49
Non-SL	0								

^a^ANCOVA model adjusted for gender, age, space closure, and treatment mechanic,.^b^binary logistic regression adjusted for gender and age, ^c^regression coefficient: average difference in number of visits / duration of treatment [in months] between the Non-SL (reference group) and the SL group (comparison group), ^d^Odds ratio: chance of extraction occurring in the SL group compared to the chance of extraction occurring in the Non-SL group. An OR of 1 indicate equal chances in both groups, OR > 1 indicate higher chance in the SL vs. non-SL group, OR < 1 indicate lower chance in the SL vs. Non-SL group, ^d^ Reference category.

CI confidence interval, OR: odds ratio, rRR: relative root resorption, sEARR: severe external apical root resorptions, SL: self-ligating.

## Discussion

The present retrospective study was intended to determine the amount of EARR, as well as the occurrence of sEARR on maxillary and mandibular incisors in a large amount of patients treated with SL or Non-SL brackets.

Extraoral x-rays such as panoramic radiographs are considered to be less precise than periapical radiographs for the determination of EARR. The main disadvantage of panoramic radiographs is an overestimation due to the magnification of the radiographs up to 20%. In the anterior region, where EARR was measured, the difference between two radiographs was observed to be less than 0.2 mm [35]. Therefore direct metric evaluations of panoramic radiographs are generally accepted as being unreliable due to the large magnification and poor reproducibility [36]. Thus, many investigators have opted to use relative values and classify their findings into resorption stages. By measuring the crown/root-ratio seemingly root shortening caused by proclination of incisors does not distort the relative values. Amongst a variety of techniques to grade EARR we chose to use the root resorption grades according to Malmgren et. al [15], as their classification has been employed in previous studies [10,13,20,27,37].

The quantity of patients in our study was in comparison to previous studies very large, giving the possibility to display a higher value of significance. Furthermore all patients were treated by one practitioner with the same bracket prescription and the same sequence of archwires, which offered a very good comparability.

The amount of EARR in the present study showed no statistically significant difference after orthodontic treatment with the SL or Non-SL brackets. Pandis et al. also did not observe a significant difference between SL and Non-SL brackets in the amount of EARR [34]. Although they compared two totally different brackets and their study displayed a small patient collective, our findings are consistent with their results. The working group of Leite et al. analyzed the amount of EARR between SL and Non-SL brackets by using cone beam computed tomography. Only 19 patients were involved in the study, but all teeth were analyzed exactly by cone beam computed tomography. However they could also not detect any significant difference between the two types of brackets [32]. As far as we know our study is based on the largest evaluated collective by now confirming previous results that the type of bracket does not have any impact on the amount of EARR.

To evaluate the occurrence of sEARR with a statistical relevance, a large amount of patients is needed due to the rare appearance. Sehr et al. managed to achieve statistical significant results about the occurrence of sEARR (grade IV according to Malmgren) in patients treated with fixed orthodontic appliances. They could show, that 0.5% of orthodontically treated teeth were affected by sEARR [27]. Thus, the 0.5% of affected teeth in the group of patients with Non-SL brackets of our study is in line with their study. As a result we could approve their findings and show for the first time, that there is no difference in the occurrence of sEARR between SL and Non-SL brackets.

In the present study the central incisors of the mandible were most often affected by sEARR. Most of the previous studies show, that the maxillary and mandibular incisors are more affected by EARR. This might be caused by higher mechanical load due to their smaller root surfaces. The observations of different studies vary equally between central or lateral incisors being more affected by EARR [8,19,20,27].

Treatment time of the two groups differed, whereby the treatment time was 2.6 months shorter with Non-SL brackets compared to SL brackets. This is in concordance with the randomized clinical trial of Fleming et al. [31]. They analyzed the same brackets as we did and also observed that duration treatment with the SL brackets was about 3 months longer. Their difference was not statistically significant due to the smaller amount of patients. Scott et al. analyzed the time for alignment between Non-SL and SL brackets. They found a slightly longer time for the SL brackets, which was also not statistically significant [38]. Due to the larger amount of patients in our study we were able to confirm their results with a statistical relevance. There are also studies describing no differences in treatment time or a faster treatment time for SL brackets [33,39,40]. Differences between the studies could be due to the use of different ligatures for Non-SL brackets. In our study Non-SL brackets were combined with steel ligatures whereas other studies use rubber elastics, which might have an impact on the friction values of the inserted archwire.

Number of appointments differed slightly between the two groups but without statistical relevance in the multivariable analysis. This finding is similar to the results of Fleming et al., who observed two more appointments for the group of patients treated with SL brackets compared to Non-SL brackets [31]. In this point the results of different studies are also contradictory, showing that the type of bracket does not influence the necessary appointments of the patients with the practitioner [28].

Companies promote a better alignment with less need of extraction when fixed appliances with SL brackets are used. We compared the number of extraction cases of both groups of patients and could not detect any difference in the number of extraction cases. Ong et al. analyzed the initial alignment of patients treated with SL and Non-SL brackets and focused on the changes of intercanine and intermolar width. SL brackets did not show a higher efficiency in initial alignment and changes of the arch dimension during treatment were similar between SL and Non-SL brackets [41]. Taken together there is no evidence that SL brackets can reduce the need for tooth extraction or improve alignment compared to Non-SL brackets.

## Conclusion

As far as we know this is the largest study by now, showing that there is no significant difference in the amount of EARR between patients treated with SL or Non-SL brackets. Furthermore this is the first study showing that there is no difference in the occurrence of sEARR between the two types of brackets.

The following conclusions can be drawn from this clinical investigation:

1.)  There was no statistically significant difference in the amount of EARR and the occurrence of sEARR between the two types of brackets.

2.)  Central mandibular incisors were mostly affected by sEARR.

3.)  Number of appointments did not display any difference between Non-SL and SL brackets, whereas treatment time with SL brackets was almost three months longer, which was statistically relevant.

4.)  There was no evidence for a difference in the amount of extraction cases in the two groups.

## Competing interests

The authors declare that they have no conflict of interests.

## Authors’contributions

CJ carried out the conception of the study. He supervised the measurements, assembled the data, conducted the interpretation of the data, and drafted the manuscript. PFG did the measurements of the panoramic radiographs. VJ was involved in conception and design of the study, also in analysis and interpretation of data, and drafting the manuscript. MH analyzed the data, did the statistics and helped with the manuscript. DM was involved in conception and design of the study, especially the correct interpretation of the radiographs. HW conceived of the study, and participated in its design and coordination and helped to draft the manuscript. All authors read and approved the final manuscript.
